# Regeneration and Endogenous Phytohormone Responses to High-Temperature Stress Drive Recruitment Success in Hemiepiphytic Fig Species

**DOI:** 10.3389/fpls.2021.754207

**Published:** 2021-11-29

**Authors:** Chuangwei Fang, Huayang Chen, Diana Castillo-Díaz, Bin Wen, Kun-Fang Cao, Uromi Manage Goodale

**Affiliations:** ^1^Guangxi Key Laboratory of Forestry Ecology and Conservation, College of Forestry, Guangxi University, Nanning, China; ^2^State Key Laboratory of Conservation and Utilization of Subtropical Agro-Bioresources, College of Forestry, Guangxi University, Nanning, China; ^3^State Key Laboratory of Vegetation and Environmental Change, Institute of Botany, Chinese Academy of Sciences, Beijing, China; ^4^Seed Conservation Specialist Group, Species Survival Commission, International Union for Conservation of Nature, Gland, Switzerland; ^5^Center for Integrative Conservation, Xishuangbanna Tropical Botanical Garden, Chinese Academy of Sciences, Beijing, China

**Keywords:** heat stress, *Ficus*, plant hormones, seed and seedling, climate change

## Abstract

Exposure to high-temperature stress (HTS) during early regeneration in plants can profoundly shape seed germination, seedling growth, and development, thereby providing stress resilience. In this study, we assessed how the timing of HTS, which was implemented as 8 h in 40°C, could affect the early regeneration stages and phytohormone concentration of four hemiepiphytic (Hs) and four non-hemiepiphytic (NHs) *Ficus* species. Their seed germination, seedling emergence, and seedling survival probabilities and the concentrations of three endogenous phytohormones, abscisic acid (ABA), indole-3-acetic acid (IAA), and salicylic acid (SA) were assessed after HTS imposed during imbibition, germination, and emergence. In both groups, seeds were more sensitive to HTS in the early regeneration process; stress experienced during imbibition affected emergence and survival, and stress experienced during germination affected subsequent emergence. There was no effect from HTS when received after emergence. Survival was highest in hemiepiphytes regardless of the HTS treatment. The phytohormones showed growth form- and regeneration stage-specific responses to HTS. Due to the HTS treatment, both SA and ABA levels decreased in non-hemiepiphytes during imbibition and germination; during germination, IAA increased in hemiepiphytes but was reduced in non-hemiepiphytes. Due to the HTS treatment experienced during emergence ABA and IAA concentrations were greater for hemiepiphytes but an opposite effect was seen in the two growth forms for the SA concentration. Our study showed that the two growth forms have different strategies for regulating their growth and development in the early regeneration stages in order to respond to HTS. The ability to respond to HTS is an ecologically important functional trait that allows plant species to appropriately time their seed germination and seedling development. Flexibility in modulating species regeneration in response to HTS in these subtropical and tropical *Ficus* species could provide greater community resilience under climate change.

## Introduction

Temperatures above the normal optimum are sensed as temperature stress by all living organisms. Plant response to high-temperature stress (HTS) during early regeneration stages determines recruitment success and the diversity and abundance of a given species with cascading effects on community composition and ecosystem function (Walck et al., 2011). Among suitable microsite conditions appropriate for seed germination, the temperature is one of the most critical drivers that alter seed viability and shape seed germination success (Walck et al., 2011). Temperature changes can affect seed dormancy and persistence in soil, preventing, delaying, or enhancing the processes and mechanisms of seed germination, seedling development, and seedling establishment (Walck et al., 2011; Chen et al., 2021). However, due to abrupt variation in climate, which is driven by anthropogenic activities, suitable temperature conditions for successful seed development and germination have been significantly altered in natural landscapes (Liu et al., 2018; Uriarte et al., 2018; Wan et al., 2018). Most often temperature stress goes hand in hand with severe drought further impeding the regeneration process due to low levels or lack of water.

Severe climate variations have led to extreme weather resulting in unprecedented phenomena such as El Niño and La Niña. Sudden changes in climate trigger drastic temperature alterations accompanied by heatwave conditions, intense rainfall, or shifts in seasonality that lead to prolonged drought and extreme flooding (Jentsch and Beierkuhnlein, 2008). While extreme weather events may be transitory, they have dramatic ecological consequences that could significantly impact ecosystem function and forest regeneration compared to just increases in average temperatures (Franklin et al., 2016). However, we have very limited knowledge on how early plant regeneration stages respond to extreme weather events, especially for forest tree species (Hou et al., 2014; Concilio et al., 2015; Luna et al., 2019). Understanding how these events may affect plant regeneration processes and future forest composition will allow us to propose suitable strategies for forest management. However, these events are very difficult to simulate under field conditions to conduct controlled experimental studies and chamber experiments provide a suitable alternative, especially for the exploration of how such extreme conditions can affect the early regeneration process.

Current scientific knowledge on how plant regeneration is affected by HTS is mostly based on studies conducted on the model plant *Arabidopsis thaliana* and a few agricultural species (Wahid et al., 2007; Toh et al., 2008; Tan et al., 2013; Quint et al., 2016; Begcy et al., 2018; Gao et al., 2020). Stress tolerance can profoundly shape seed germination, seedling growth, and development, metabolism, and physiology (Han et al., 2019). Seed germination requires suitable temperatures for breaking the dormancy and initiating biochemical mechanisms, such as protein and plant hormones mobilization and activation (Miransari and Smith, 2014; Gao et al., 2017; Vishwakarma et al., 2017; Agostinetto et al., 2018). When seeds face HTS, higher probabilities of seed mortality, slow germination rates, and germination failure will occur (Weitbrecht et al., 2011; Tan et al., 2013; Tangney et al., 2019) and the resulting seedlings will be less resilient due to low stored resources and more vulnerable to future stressors due to impaired development of shoot and root growth (Uriarte et al., 2018).

Under conditions of environmental stress, plants are able to adapt and regulate their growth through the production of specific phytohormones, which influences division and differentiation of cells and plant regeneration, thus providing stress resilience (Peleg and Blumwald, 2011; Fahad et al., 2014; Quint et al., 2016). The relationship between stresses and plant hormones has shown that diverse plant species have specific responses at different regeneration stages under HTS. Plants can regulate the level of hormones, and therefore metabolism, during regeneration through stress signaling related to specific gene expression (Wahid et al., 2007; Verma et al., 2016; Zhu, 2016). Sensing and stress signaling are two of the most important responses that plants have evolved in response to elevated temperatures that minimize damage and ensure the protection of cellular homeostasis. Both plant hormones and reactive oxygen species also contribute to temperature stress signaling, which disturbs cellular homeostasis that can lead to severe retardation in growth and development, and finally impact plant survival (Kotak et al., 2007).

Phytohormones are good candidates to assess HTS resilience in plants as they interact during plant growth and play a critical role in providing strategies for plant adaptation to environmental stress. Phytohormones such as abscisic acid (ABA), indole-3-acetic acid (IAA), cytokinins, gibberellins (GAs), ethylene, brassinosteroids (BRs), jasmonic acid (JA), and salicylic acid (SA) regulate pivotal functions in different physiological and biochemical plant processes (Suzuki et al., 2014). The phytohormone ABA, a sesquiterpene, which controls several developments and growth processes of plants such as leaf abscission, and inhibition of fruit ripening (Vishwakarma et al., 2017) is considered as the “stress hormone” that responds to a diversity of biotic and abiotic stressors including heavy metal, drought, salinity, temperature, and radiation stress (Zhang, 2014). It can specifically affect plant regeneration through extending seed dormancy (Suzuki et al., 2014) and inhibiting seed germination by delaying the radicle expansion and weakening of endosperm, as well as enhancing expression of transcription factors (Graeber et al., 2010). The response to environmental stresses is also driven by IAA (Zhao et al., 2020), an essential hormone for the process of somatic embryogenesis, which plays a key role in regulating cell cycling, formation of vascular tissues and pollen, governing seedling growth, and embryo, leaf, and root development (Suzuki et al., 2014). IAA and brassinosteroids hormones have the ability to stimulate ethylene production, and along with ABA can induce seed germination by rupturing testa and endosperm, while ABA alone has the opposite effects on seed germination (Suzuki et al., 2014). Phenolic phytohormones, such as salicylic acid (SA), mediate plant responses to abiotic stresses such as drought, chilling, heavy metal toxicity, heat, and osmotic stress (Rivas-San Vicente and Plasencia, 2011) as well as against biotic stresses such as pathogens and pest attacks (Bari and Jones, 2009). The role of SA in seed germination has been controversial. There are conflicting reports suggesting that it can either inhibit germination (Rajjou et al., 2006; Xie et al., 2007) or increase seed vigor under different abiotic stress conditions (Rajjou et al., 2006; Alonso-Ramírez et al., 2009), which has been linked to the concentration of SA applied exogenously in experimentation (Rivas-San Vicente and Plasencia, 2011). This phenolic compound has a wide range of distribution in plants, as well as various levels of expression among species and plays a key role during photosynthesis, transpiration, and ion uptake and transport (Alonso-Ramírez et al., 2009). However, the contribution of phytohormones to early plant regeneration processes is mainly studied in mature plants and specifically in the model plant, *A. thaliana* (Miransari and Smith, 2014; Lymperopoulos et al., 2018).

Species with a wide variety of ecological attributes such as the members of the genus *Ficus*, commonly known as figs, provide a good model system to understand the mechanisms that underlie the environmental response and endogenous phytohormones expression during the early regeneration process (Hao et al., 2010, 2011a, 2013; Jin et al., 2015; Castillo-Díaz et al., 2021; Chen et al., 2021). *Ficus* comprises more than 750 species in diverse life forms such as shrubs, vines, lianas, hemiepiphytes, and non-hemiepiphytes (Berg, 1989; Harrison et al., 2003; Harrison and Shanahan, 2006). Hemiepiphytes, which make up the half of *Ficus* species characteristically start their life as epiphytic plants in the forest canopy and send their areal roots to the ground to become terrestrial plants as adults (Harrison et al., 2003), while non-hemiepiphytic species begin life on the forest floor and become mature adults and continue their terrestrial growth form. These contrasting growth forms that accompany different eco-physiological mechanisms are a consequence of environmental adaptations resulting in strong evolutionary changes, making this genus an ideal model system for comparative plant eco-physiological studies (Harrison, 2005; Harrison and Shanahan, 2006; Hao et al., 2013; Castillo-Díaz et al., 2021; Chen et al., 2021). Many figs are keystone species that have a relatively low abundance compared to their influence on the ecosystem (Paine, 1969; Mills and Doak, 1993; Power et al., 1996). Thus, their loss could cause a disproportionately large effect on communities as they fulfill important functions in biological and ecological interactions (Mills and Doak, 1993; Harrison, 2003; Seekar et al., 2010; Peabotuwage et al., 2019). Despite their importance, we have limited knowledge on the responses of plant hormones to temperature stress during the early regeneration of *Ficus*.

In this study, we assessed the effects of temperature stress on the early regeneration process (seed germination, seedling emergence, and seedling survival) and three endogenous phytohormones, namely indole-3-acetic acid (IAA), abscisic acid (ABA), and salicylic acid (SA), of eight *Ficus* species after subjecting them to temperature stress during seed imbibition to seedling development. Using both hemiepiphytic (Hs) and non-hemiepiphytic congeneric species (NHs), we tested the following hypotheses: (1) hemiepiphytic species will have greater resilience to temperature stress and a higher probability of successful germination, seedling emergence, and survival compared to non-hemiepiphytes. Previous studies have shown the evolutionary strategies developed by hemiepiphytic species to face and overcome a variety of environments resulting in greater germination and seedling survival (Hao et al., 2011a; Chen et al., 2021). (2) The concentration of ABA would be higher during seed germination under greater temperature stress in both groups but the change would be greater for hemiepiphytic species. (3) The phytohormone IAA concentration would be higher during germination and decrease with the seedling emergence, with greater secretion when plants are subjected to temperature stress, especially in hemiepiphytic species. (4) We also expect that SA concentrations will be markedly higher in hemiepiphytic species under greater temperature stress during seed germination and seedling emergence.

## Materials and Methods

### Study Species and Seed Collection

Mature syconia (i.e., fruits) of four non-hemiepiphytic and four hemiepiphytic *Ficus* species studied here (Table 1; Chang et al., 1998; Zhou and Gilbert, 2003; Berg, 2004; Cruaud et al., 2012) were randomly collected from Yunnan and Guangxi provinces, southern China from 2019 to 2020 and identified to the species level. Seeds were extracted and transported to the Regeneration Ecology, Seed Biophysiology, and Conservation Laboratory at Guangxi University (Chen et al., 2021). At the laboratory, air drying was conducted in climate chambers at 25°C for a day, until seeds achieved 15% relative humidity (Hay and Probert, 2013) and then stored in paper bags at 10°C for use in future experiments.

**TABLE 1:** Characteristics of four hemiepiphytic (H) and four non-hemiepiphytic (NH) *Ficus* species investigated in this study*.

**Species**	**ID**	**Adult growth form (max height in m)**	**Habitats**	**Elevation (m a.s.l)**	**Distribution in China**	**Growth form**	**Subgenus**	**Section**
*Ficus auriculata* Lour.	FIAUR	Trees, (4–10)	Forests in moist valleys	100–2100	S Guangdong, Guangxi, Hainan, SW Guizhou, SW Sichuan, Yunnan	NH	*Sycomorus*	*Neomorphe*
*Ficus oligodon* Miq.	FIOLI	Trees, (5–10)	Valleys, along streams, moist soil areas	200–2100	Guangxi, Guizhou, Hainan, SE Xizang, Yunnan	NH	*Sycomorus*	*Neomorphe*
*Ficus racemosa* L.	FIRAC	Trees, (25–30)	Moist areas, beside rivers and streams, and occasionally in streams	100–1700	S Guangxi, Guizhou, Yunnan	NH	*Sycomorus*	*Sycomorus*
*Ficus semicordata* Buch.*-Ham.ex Sm*.	FISEM	Trees, (3–10)	Forest margins, valleys, along trails	600–2800	Guangxi, Guizhou, SE Xizang, Yunnan	NH	*Sycomorus*	*Hemicardia*
*Ficus benjamina* L.	FIBEN	Trees, (20)	Moist mixed forests	500–800	SW Guangdong, Guangxi, Guizhou, Hainan, S Taiwan, Yunnan	H	*Urostigma*	*Conosycea*
*Ficus concinna* (Miq.) Miq.	FICON	Trees, (15–20)	Dense forests and near villages	900–2400	Fujian, Guangdong, Guangxi, Guizhou, S Jiangxi, SE Xizang, Yunnan, S Zhejiang	H	*Urostigma*	*Urostigma*
*Ficus microcarpa* L.f.	FIMIC	Trees, (15–25)	Mountains and plains	Below 1900	Guangdong, Guangxi, Guizhou, Hainan, Taiwan, Yunnan, S Zhejiang	H	*Urostigma*	*Conosycea*
*Ficus religiosa* L.	FIREL	Trees, (15–25)	Cultivated	Low to high elevations (mostly cultivated)	Guangdong, Guangxi, S Yunnan	H	*Urostigma*	*Urostigma*

*Information obtained from Flora of China (Chang et al., 1998; Zhou and Gilbert, 2003) and phylogeny published by Berg (2004) and Cruaud et al. (2012).

### Seed Viability Assessment

Before experimentation, seeds were further cleaned to remove any debris and empty seeds were separated from filled seeds by fanning. Subsamples of all seed lots were tested for viability (Chen et al., 2021) by germinating in Petri dishes containing 18 g of 1,300 g cm^–2^ agar (Coolaber, Beijing Cool Technology Co., Beijing, China) dissolved in 1 l of deionized water. We placed the germination test in HTR-3X100 germination chambers (He Tian Equipment Co. Ltd., Shanghai, China) under 12 h light and 12 h dark, day and night conditions, ∼60% relative humidity, 25°C day and 15°C night temperature with photosynthetic photon flux density (PPFD) 400 μmol m^–2^ s^–1^ emitting visible light, i.e., 400–700 nm. Seed lots with > 98% viability were surface sterilized (1% sodium hypochlorite solution for 3 min) and used for the following experiments.

### Assessment of the Temperature Stress Effect on the Early Regeneration

**FIGURE 1:**
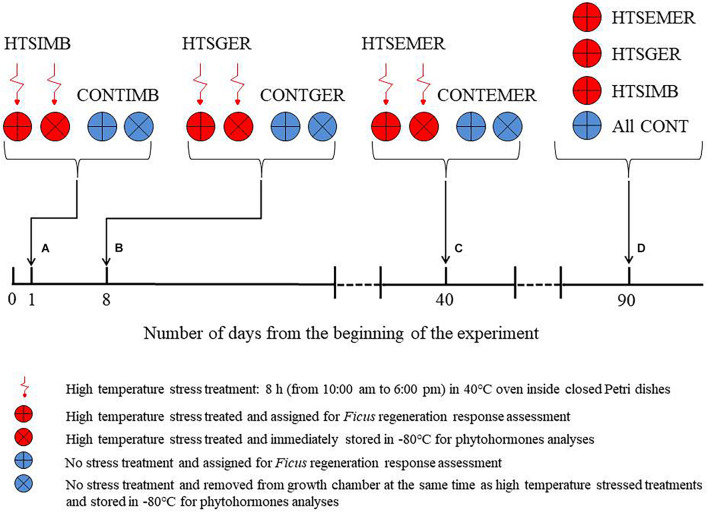
Diagrammatic representation of experimental design indicating the timeline for administering the high-temperature stress (HTS) treatment by placing Petri dishes in 40°C temperature stress treatment for 8 h from 10:00 to 18:00 in a conventional oven: **(A)** 24 h after the experiment began during the time seeds undergo imbibition (IMB), **(B)** 8 days after the experiment began during seed germination (GER), and **(C)** 40 days after the experiment began during seedling emergence (EMER). We implemented the 40°C temperature stress treatment at each level just once in the duration of the experiment. All temperature stress-treated Petri dishes with seeds or seedlings were returned to the growth chamber at the end of each treatment. Each HTS treatment had a control (CONT) at 25/15°C day/night temperature and thus, we had a total of 512 Petri dishes and six treatment conditions (HTSIMB and CONTIMB, HTSGER and CONTGER, and HTSEMER and CONTEMER). The experiment was concluded in 90 days **(D)**. There were additional 192 Petri dishes that were assigned to destructive sampling for the phytohormone analysis.

Using approximately 136,340 seeds of eight *Ficus* species, we assessed the effect of 40°C temperature stress on *Ficus* seed germination, seedling emergence, and seedling survival and three endogenous phytohormones, i.e., IAA, ABA, and SA. We used 50 mm diameter Petri dishes for regeneration observation and 100 mm diameter Petri dishes for phytohormones identification as greater space for seedlings growth was needed (Figure 1). Four Petri dishes per species and 20 seeds in each Petri dish on agar medium were used and randomly assigned in four germination chambers under the aforementioned conditions.

The 40°C temperature stress treatment consisted of three regeneration stages that were timed based on prior experiments (Chen et al., 2021): (1) 24 h after the experiment began during the time seeds undergo imbibition (IMB), (2) 8 days after the experiment began during seed germination (GER), and (3) 40 days after the experiment began, during seedling emergence (EMER). We implemented the 40°C temperature stress treatment at each level just once in the duration of the experiment, i.e., if we submitted the seeds under 40°C temperature stress during imbibition we did not submit them again for any other heat stress treatment during subsequent regeneration of the same seeds. Each level of the treatment was subjected to temperature stress by removing Petri dishes with seeds from the growth chamber and placing them in 40°C for 8 h from 10:00 am to 6:00 pm in a conventional oven (Sobo 101-WB, Shaoxing Sobo Instrument Co., Ltd, Shaoxing, China). All temperature stress-treated Petri dishes with seeds or seedlings were returned to the growth chamber at the end of each treatment. Each HTS treatment had a control (CONT) at 25/15°C day/night temperature and thus, we had a total of 512 Petri dishes and six treatment conditions (HTSIMB and CONTIMB, HTSGER and CONTGER, HTSEMER, and CONTEMER; Figure 1).

We assessed the regeneration responses following Chen et al. (2021) as follows. A *Ficus* seed that successfully reached or passed each life stage was scored as a successful event and an individual that failed to reach or pass the same event was scored as a failure. We assessed *Ficus* seed germination as 2 mm radicle emergence and cotyledon formation as seedling emergence every day for the first 30 days, once every 7 days for the next 60 days, and then at the end of the experiment at 90 days. Seedlings were considered as survived if cotyledons turned green and did not wither or die until the end of the experimental period. We considered dead seedlings as those that had dried, decayed, or become gray or black in color and remained so for 20 days after completing the experiment without any growth or greening.

### Assessment of the Temperature Stress Effect on the Phytohormone Responses

For the phytohormones assessment (Supplementary Figure 1), we used four additional replicates assigned for destructive sampling, in both control and temperature stress treatment Petri dishes. These replicates with a total of 192 were sown with 0.2 g seeds of each species, with two Petri dishes in each replicate treatment. Immediately after each temperature stress treatment, both temperature stress treated and control sample plant materials from the additional replicates were collected into 5 ml centrifuge tubes and stored in the in −80°C until prepared for phytohormone analysis (Fu et al., 2012; Sheflin et al., 2019; Cao et al., 2020).

Fresh plant material stored for phytohormone analyses were smashed into powder using a freezer mixer (HF-64LD, He Fan Instrument Co., Ltd, Shanghai, China) at −20°C, 60 Hz for 30 s and 0.15 g of the ground sample was added to a centrifuge tube containing 0.5 ml of extraction solution made with a volume ratio of 75:20:5 of methanol, ultrapure water, and formic acid (HPLC grade, Thermo Fisher Scientific, United States) and 0.05 g polyvinylpolypyrrolidone (PVPP, Solarbio, Beijing, China; Fu et al., 2012) and homogenized using a vortex mixer for 30 s (MIX-VR, Tuohe Electromechanical Technology Co., Ltd, Shanghai, China). Then, samples were further mixed using an ultrasonic machine (40KHz, JP-100X, Skymen Equipment Co., Ltd, Shenzhen, China) for 30 min under ice water, and were stored at 4°C for 16 h. Afterward, 1 ml methylene dichloride (HPLC grade, Thermo Fisher Scientific, United States) was added to every sample and mixed using an ultrasonic machine for 30 min under ice water. Samples were centrifuged (12,000 rpm at 4°C for 15 min) and the subnatant was separated and dried under nitrogen gas (Fu et al., 2012; Sheflin et al., 2019). They were then dissolved using 500 μl 50% methanol (high-performance liquid chromatography [HPLC] grade) and 1% formic acid (HPLC grade) and were further purified using 0.22 μm nylon needle filter and transferred to new vials with glass insert and stored at −80°C until ultra-performance liquid chromatography-tandem mass spectrometry (UPLC-MS/MS, EXPEC-5210, Expec Technology Co., Ltd, China) analysis of phytohormones ABA, IAA, and SA (detailed procedures and parameters for setting equipment during phytohormone measure are given in Supplementary Tables 1–4).

### Statistical Analysis

We conducted all statistical analyses using *glmmTMB* and *lme4* packages in R version 4.0.3 (Bates et al., 2015; Brooks et al., 2017; R Core Team, 2019), and all figures were generated using the *ggplot 2* packages (Wickham, 2009). From binary response variables using the *cbind* function in R, we calculated the probability of seed germination, seedling emergence, and seedling survival as follows. For example, we defined “1 = survived at the end of the 90-day experimental period” and “0 = failed to survive at the end of the 90-day experimental period” (Chen et al., 2021). The probability of cumulative seed germination, seedling emergence, and seedling survival was calculated as the ratio among the number of germinated seeds, emerged seedlings, and survived seedlings and the total number of failures during the 90-day experimental period. Probability data are presented as values between 0 and 1. To assess the effect of the temperature stress treatment and the two contrasting growth forms on seed germination, seedling emergence, and seedling survival, we used them as categorical fixed effects and species as a random factor in generalized linear mixed models, assuming a binormal distribution using a log-link function (Sileshi, 2012; Hay et al., 2014; Chen et al., 2021) in the following model:


$$Y∼\beta_{0}+\beta_{1}\,T\,S\,T+\beta_{2}\,G\,F+\beta_{3}\,T\,S\,T\,\_\,G\,F+\varepsilon_{s\,p}+\varepsilon_{r\,e\,s\,i\,d\,u\,a\,l}$$

where, *Y* is the early regeneration response (seed germination, seedling emergence, and seedling survival), *TST* represents the temperature stress treatment, *GF* represents growth form, *TST_GF* represents the interaction between temperature stress treatment and growth form, and ε*_*sp*_* and ε*_*residual*_* represent the species as a random factor and the residual error, respectively. We assessed the best-fit model among the binomial model, observation-level random-effects model, and the Beta-binomial model using Akaike’s information criterion (ΔAIC) values. We found that the Beta-binomial model, which quantifies and models the excess variation from overdispersion, best explained the variation in our data (Harrison, 2015).

To assess the effect of the temperature stress treatment and growth form on three phytohormones: ABA, IAA, and SA, we used them as categorical fixed effects and species as a random factor in linear mixed models, assuming a normal distribution in the following model:


$$Y∼\beta_{0}+\beta_{1}\,T\,S\,T+\beta_{2}\,G\,F+\varepsilon_{s\,p}+\varepsilon_{r\,e\,s\,i\,d\,u\,a\,l}$$

where, *Y* is the phytohormones response (ABA, IAA, and SA concentration), *TST* represents the temperature stress treatment, *GF* represents growth form, and ε*_*sp*_* and ε*_*residual*_* represent species as a random factor, and the residual error, respectively.

All models were evaluated using the variance inflation factor (VIF) < 2 for collinearity among model predictor variables using the *car* package (Fox and Weisberg, 2019). We evaluated model fit the full dataset using marginal and conditional *R*^2^ values (*R*^2^ marginal and *R*^2^ conditional, respectively) developed for mixed effect models (Nakagawa and Schielzeth, 2013). Within the context of each fitted full model, the influence of fixed factors was tested using the *car* package.

## Results

The assessment of the effect of temperature stress during seed imbibition, seed germination, seedling emergence on the early regeneration stages (seed germination, seedling emergence, and seedling survival) of the eight *Ficus* species showed that *Ficus microcarpa* had the highest survival under all temperature stress treatments (Supplementary Figure 2). Seedling survival was highest for hemiepiphytic species (47.35 ± 0.27%) when the temperature stress treatment was experienced during seedling emergence and seedling survival was lowest for the same treatment for non-hemiepiphytic species (26.18 ± 0.16%).

### Effect of Temperature Stress Experienced During Seed Imbibition

**FIGURE 2:**
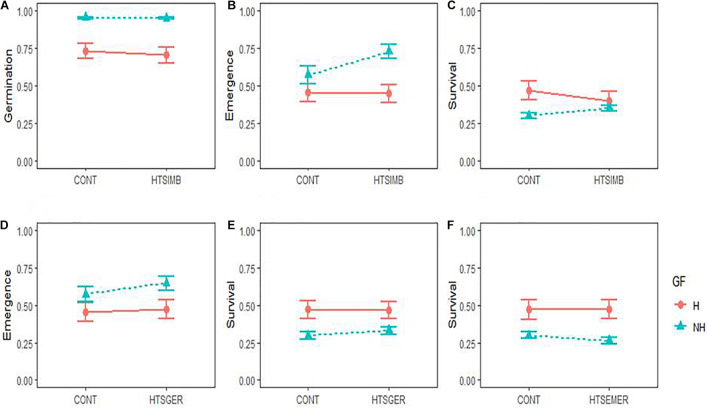
Early regeneration responses in two growth forms (GF; H = hemiepiphytic and NH = non-hemiepiphytic) of *Ficus* species to high temperature stress (HTS) applied at different regeneration stages: seed imbibition (HTSIMB), seed germination (HTSGER), and seedling emergence (HTSEMER). The HTS was applied as 40°C for 8 h from 10:00 am to 6:00 pm, and the respective control treatment (CONT) was not given an HTS treatment. The responses to HTSIMB were measured as the probability of seed germination **(A)**, probability of seedling emergence **(B)**, and probability of seedling survival **(C)**. The responses to HTSGER were measured as the probability of seedling emergence **(D)** and the probability of seedling survival **(E)**. The response to HTSEMER was measured as the probability of seedling survival **(F)**.

When *Ficus* seeds were subjected to the temperature stress treatment during imbibition, although the germination of both growth forms was not affected by the temperature stress (*P* > 0.05; Table 2 and Figure 2A), non-hemiepiphytic species had 23.47 ± 0.2% more germination in both the stressed and non-stressed seeds. However, the hemiepiphytic and non-hemiepiphytic species responded differently under temperature stress experienced during seed imbibition (Figures 2A–C). There was a significant increase in seedling emergence of non-hemiepiphytic species (*P* < 0.0001; Table 2 and Figure 2B). In contrast, the seeds of the hemiepiphytic species that underwent temperature stress showed lower survival compared to control seeds and the non-hemiepiphytes showed the opposite trend (*P* < 0.0001; Table 2 and Figure 2C). Therefore, although temperature stress during imbibition affected survival, there was no effect from growth from with species from both growth forms surviving on average 37.42 ± 0.2%.

**TABLE 2:** Chi-square and *P* values for the effects of the high-temperature stress treatment (HTS) on the early regeneration probability (Ger, Emer, and Sur) and phytohormone response (ABA, IAA, and SA) and the interaction by growth form.

**Experiment factor**	**Ger_IMB**		**Emer_IMB**		**Sur_IMB**		**Emer_GER**		**Sur_GER**		**Sur_EMER**	
	**χ^2^**	***P***	**χ^2^**	***P***	**χ^2^**	***P***	**χ^2^**	***P***	**χ^2^**	***P***	**χ^2^**	***P***
High temperature stress treatment during regeneration (HTS)	2.9183	0.0835	35.4517	0.685	0.5845	<**0.0001**	13.9551	0.3192	1.3215	0.824	1.8115	0.854
Growth form (GF)	3.6924	0.0640	1.4653	0.503	0.7490	0.167	0.8775	0.4547	1.6318	0.154	2.1154	0.192
HTS*GF	0.2223	0.6373	47.8598	<**0.0001**	26.0477	<**0.0001**	5.0464	**0.0247**	2.0665	0.151	2.4716	0.116

**Experiment factor**	**ABA_IMB**		**IAA_IMB**		**SA_IMB**		**ABA_GER**		**IAA_GER**		**SA_GER**	
	**χ^2^**	***P***	**χ^2^**	***P***	**χ^2^**	***P***	**χ^2^**	***P***	**χ^2^**	***P***	**χ^2^**	***P***

High temperature stress treatment during regeneration (HTS)	0.7036	0.4016	0.4880	0.4848	7.8112	**0.0052**	7.8247	**0.0052**	0.2595	0,6104	0.9067	0.3410
Growth form (GF)	2.1193	0.1455	3.1557	0.0757	13.6530	**0.0002**	1.6668	0.1967	3.2534	0.0713	2.9999	0.0833
HTS*GF	1.2114	0.2710	0.0333	0.8553	2.9253	0.0872	5.5731	**0.0182**	5.0891	**0.0241**	0.1125	0.7373

**Experiment Factor**	**ABA_EMER**		**IAA_EMER**		**SA_EMER**							
	**χ^2^**	***P***	**χ^2^**	***P***	**χ^2^**	***P***						

High temperature stress treatment (HTS)	14.1376	**0.0002**	8.4047	**0.0037**	0.1260	0.7227						
Growth form (GF)	10.4870	**0.0012**	0.0930	0.7604	52.7154	<**0.0001**						
HTS*GF	7.3903	**0.0066**	0.4107	0.5216	2.3657	0.1240						

*The regeneration response probabilities are expressed as Ger_IMB, seed germination probability when exposed to HTS in seed imbibition stage; Emer_IMB, seedling emergence probability when exposed to HTS in seed imbibition stage; Sur_IMB, seedling survival probability when exposed to HTS in seed imbibition stage; Emer_GER, seedling emergence probability when exposed to HTS in seed germination stage; Sur_GER, seedling survival probability when exposed to HTS in seed germination stage; Sur_EMER, seedling survival probability when exposed to HTS in seedling emergence stage. Similarly, the concentration of each phytohormone (ABA, IAA, and SA) is given for each stage connected by a hyphen. Responses depicted in bold are significant at *P* < 0.05.*

### Effect of Temperature Stress Experienced During Seed Germination

When seeds were submitted to temperature stress during seed germination, we found 17.43 ± 0.27% greater seedling emergence in non-hemiepiphytic species but with greater survival for hemiepiphytic species. However, temperature stress during germination only affected the emergence of non-hemiepiphytes with a 7.58 ± 2.24% increase in emergence (*P* = 0.0247; Table 2 and Figure 2D). There was no significant effect from temperature stress for the survival of either group (Table 2 and Figure 2E).

### Effect of Temperature Stress Experienced During Seedling Emergence

Here, as it was observed for seedling survival for the seeds subjected to temperature stress during imbibition and germination, hemiepiphytic species showed greater survival in both the control and treated seeds (17.28 ± 0.25% and 21.17 ± 0.25%, respectively). Seeds of both groups subjected to temperature stress during seedling emergence showed no effect for their subsequent survival (Table 2 and Figure 2F).

### Effect of Temperature Stress on Phytohormones

**FIGURE 3:**
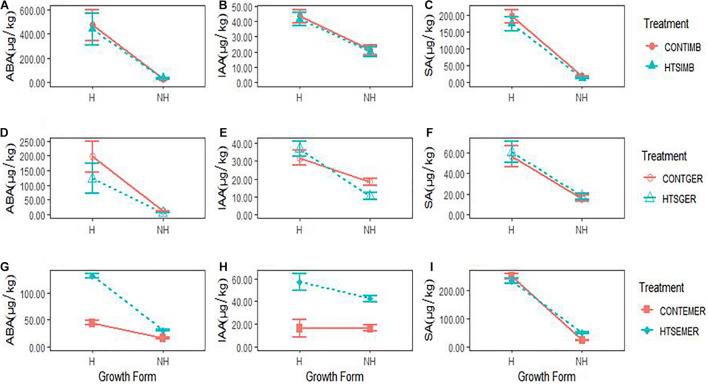
The concentrations of three endogenous phytohormones, abscisic acid (ABA), indole-3-acetic acid (IAA), and salicylic acid (SA), in two growth forms (H = hemiepiphytic and NH = non-hemiepiphytic) of *Ficus* species when high temperature stress (HTS) was applied at different regeneration stages: seed imbibition (HTSIMB), seed germination (HTSGER), and seedling emergence (HTSEMER). The HTS was applied as 40°C for 8 h from 10:00 am to 6:00 pm, and the respective control treatment (CONT) was not given an HTS treatment. **(A–C)** The concentration of ABA, IAA, and SA after HTSIMB and for their control treatments graphs. **(D–F)** The concentration of ABA, IAA, and SA after HTSGER and for their control treatments. **(G–I)** The concentration of ABA, IAA, and SA after HTSEMER and for their control treatments.

The phytohormones showed growth form- and regeneration stage-specific responses to HTS (Figures 3A–I and Supplementary Figure 3). The ABA concentration expressed during this study was highest during seed imbibition of hemiepiphytic species, which was greater than non-hemiepiphytic species under all treatment conditions (Figures 3A,D,G). The IAA concentration was also on average greater for hemiepiphytic species but more than twofold greater for the HTS treated and control plants when assessed for emerged seedlings (Figures 3B,E,H). SA concentration was higher for hemiepiphytic species compared to non-hemipephytes under all treatments, but with no significant difference between high temperature treated and control plants (Figures 3C,F,I). When the temperature stress treatment was imposed during seed imbibition, of the three phytohormones assessed in our study, only the SA levels were significantly affected due to the temperature stress treatment, which declined in non-hemiepiphytic species (both *P* = 0.0052; Table 2 and Figures 3A–C). During germination, the temperature stress received by seeds affected ABA as well as IAA concentrations (Figures 3D–F). Similar to the SA during imbibition, the ABA concentration declined significantly in non-hemiepiphytic species (*P* = 0.0182; Table 2 and Figure 3D). However, in the case of IAA, the two growth forms displayed contrasting effects; the effect of temperature stress on germinating seeds was seen to increase IAA in hemiepiphytic species but was reduced in non-hemiepiphytic species (*P* = 0.0241; Table 2 and Figure 3E). When regeneration progressed from germination to emerging seedlings and they were subjected to the temperature stress treatment, all three phytohormones showed significant effects due to the stress treatment. Temperature stress imposed on emerging seedlings affected ABA by a threefold increase in hemiepiphytic species and the non-hemiepiphytic species showed less than onefold increase (*P* = 0.0066; Table 2 and Figure 3G). Both hemiepiphytic and non-hemiepiphytic species showed significant increases in IAA when their emerging seedlings were exposed to the temperature stress treatment with the effect on hemiepiphytic species being greater compared to the non-hemiepiphytic species (*P* = 0.0037; Table 2 and Figure 3H). In contrast, when emerged seedlings were exposed to the temperature stress treatment, hemiepiphytic species did not show any significant differences in the SA between stress treated and controlled seedlings but the non-hemiepiphytic species showed an increase in SA (*P* < 0.0001; Table 2 and Figure 3I). The phytohormone concentration was significantly greater in hemiepiphytic species (Figures 3A–J) except for IAA when the temperature stress treatment was imposed on emerging seedlings (Figure 3I).

## Discussion

During natural regeneration transient stress conditions can impose physiological barriers that can drastically inhibit growth limiting seed germination, seedling emergence, and, finally, survival. Temperature is one of the most important abiotic factors that can influence the successful transition from one early regeneration stage to another (Baskin and Baskin, 2014a). The interaction of temperature with other inherent biotic conditions, such as phytohormones and plant growth form, can further modulate the regeneration success and recruitment into the next life-history stage (Chen et al., 2021). How the early regeneration stages of contrasting growth forms respond to temperature stress is an important fundamental investigation in plant sciences. In this study, we provide detailed evidence of how temperature stress affects the early regeneration process and phytohormones in eight *Ficus* species belonging to two divergent growth forms. Our results show that in both hemiepiphytic and non-hemiepiphytic species, seeds are more sensitive to temperature stress when stress is experienced early in the regeneration process, with stress experienced during imbibition affecting emergence and survival and stress experienced during germination affecting emergence. There was no effect from the temperature stress if it was received after seedling emergence. Thus, our detailed empirical study shows that environmental variability affects the transition from on early regeneration stages to the next (Clark et al., 1998).

Temperature variations significantly impact plant regeneration, altering forest structure and composition (Christian, 2001; D’Antonio et al., 2001; Seabloom et al., 2003; Hou et al., 2014). The importance of how temperature stress affects plant regeneration has only become even greater as temperatures warm due to climate change making temperature a major abiotic stress factor for plant growth (Grant et al., 2017; Liu et al., 2018; Uriarte et al., 2018; Wan et al., 2018). The stress experienced during regeneration can have cascading effects altering plant species composition, richness, and community composition, significantly altering or damaging the functions of the original ecosystem (Yan et al., 2015). In our study, when temperature stress was applied during seed imbibition and seed germination, we found positive effects on seedling emergence for non-hemiepiphytic species but seedling survival probabilities were higher for hemiepiphytic species. These contrasting advantages can be a priority effect favoring both growth forms with faster seedling development and recruitment (Alexander et al., 2015; Catelotti et al., 2020).

Prior studies have identified unique evolutionary characteristics and clear differences in the functional traits between the hemiepiphytic and non-hemiepiphytic species in the genus *Ficus* (Hao et al., 2013; Castillo-Díaz et al., 2021; Chen et al., 2021). We found that temperature stress during seed imbibition and seed germination had no significant effect on latter regeneration stages such as seed germination, seed emergence, and seedling survival of hemiepiphytes. These results indicate that seeds and seedlings of hemiepiphytic species had better stress tolerance during the early regeneration stages with more seedlings surviving the stress condition compared to non-hemiepiphytes when exposed to temperature stress, consistent with previous studies (Hao et al., 2013; Chen et al., 2021). Thus, this result partially supports our first hypothesis, since hemiepiphytic germination and emergence were not significantly different between the two groups. The resistance mechanism of abiotic stress in hemiepiphytes resulting in greater seedling survival may help them become more competitive when faced with temperature stress leading to an increase in the colonization and re-assembly of the hemiepiphytic *Ficus* species under drier and warmer canopy conditions. This scenario might be equally positive for seedling emergence of non-hemiepiphytic species when faced with temperature stress. The ability to conserve water from early regeneration (Chen et al., 2021) to sapling and tree size classes (Hao et al., 2011a,b, 2013) has provided a greater advantage for hemiepiphytic species, which is crucial for survival in drought conditions that can be important under canopy conditions. In germination chamber experiments conducted by Chen et al. (2021), on 15 *Ficus* species, hemiepiphytic species were more resilient to drought conditions and germination peaks at 25/15°C, day and night temperature but seedling survival decreased when the day and night temperature were increased to 35/25°C. This is consistent with what we observed in our temperatures stress treatment which was provided as a HTS event lasting 8 h. Our study provides further evidence that temperature stress imposes greater limitations on the early regeneration stages of these species and the response is significantly modulated by phytohormones.

Several environmental factors, particularly variations in temperature, are considered significant drivers of the early regeneration process of *Ficus* species. Temperature changes significantly influence the germination of *Ficus benjamina* L. var. *nuda* seeds, and the optimum germination temperature for this species was between 20 and 35°C or 30°C/20°C (Fu et al., 2008). *Ficus* species present a variety of germination responses under the effects of temperature as well as red:far-red light because of their different habitat preferences (Chen et al., 2013). The extremely high temperature at 51–53°C has resulted in irreversible leaf damage on potted seedlings of *Ficus insipida* Willd, a neotropical pioneer tree species (Krause et al., 2010); even though they could survive and develop new leaves under heat stress, temperatures higher than 51–53°C led to significant damage to the canopy due to lower heat acclimation capacity. Our study, one of the few conducted to explore the effect of temperature stress on specific stages of the early regeneration process, shows how the temperature stress, received for a short duration simulating a heatwave and the timing of this stress event, could affect the early regeneration stages influencing their downstream demographic processes through modulating seedling survival.

Growth form is an important factor that underpins the ecosystem structure and diversity (Rowe and Speck, 2005). The impact of temperature stress on the successful transition through each early regeneration stage can explain *Ficus* rarity as well as co-existence (Harrison, 2006; Chen et al., 2021). We did not see strong effects on germination and emergence of both growth forms when seeds were subjected to temperature stress in the first 24 h period during which imbibition takes place. However, seedling survival was affected indicating that there is a carryover effect for the latter regeneration stages from the temperature stress exposure during seed imbibition. Seed imbibition controls seed dormancy and seed germination (Preston et al., 2009) and it is an important driver of water uptake, including passive water uptake by the dry seeds, negligible water uptake, and seed germination and seedling growth (Finch-Savage and Leubner-Metzger, 2006). Here we show that under temperature stress, seed imbibition was very resilient, and thus, a highly significant factor determining the stability of metabolic activities that result in seed germination. The importance of the relative contribution from each early regeneration stage can be used to identify the most important stage that contributes to an individual fitness (Eriksson and Ehrlén, 2008). In both experimental and field studies, it has been shown that recruitment limitation became progressively higher as the *Ficus* species move forward from one early regeneration stage to the next, and environmental filters were most influential during the seedling emergence stage which creates a niche bottleneck when transitioning from an emerging seedling to become an established seedling (Castillo-Díaz et al., 2021; Chen et al., 2021). Our study shows that events occurring very early in regeneration have cascading effects on the latter regeneration stages.

In our study, the endogenous phytohormones, ABA, IAA, and SA, showed the growth form-specific responses to the temperature stress in different regeneration stages. In contrast to our second hypothesis, ABA concentration was greater for imbibition compared to germination or emergence. However, many studies would not differentiate between two stages considering seed imbibition the first step toward germination. Hence, it can be considered that the germination stage showed the greatest increase in ABA concentration. Among the phytohormones in our study, ABA and IAA were the major phytohormones to mediate temperature stress and SA was less sensitive. Consistent with our third hypothesis, the ABA levels declined significantly in non-hemiepiphytes following exposure to temperature stress during germination and it increased by threefold in hemiepiphytes and onefold in non-hemiepiphytes when temperature stress was experienced on emerging seedlings. Our results are in contrast to what was observed in imbibed *A. thaliana* seeds, where ABA levels were elevated at high temperatures (Toh et al., 2008). With IAA the two growth forms presented contrasting results for temperature stress experienced during germination, with increases observed for hemiepiphytes and decreased concentrations observed for non-hemiepiphytes. When stress was experienced by emerging seedlings both groups of species showed increased concentrations for both ABA and IAA. Physiological studies with excised stem segments have implicated that IAA regulates cell elongation (Cleland, 2010). However, supporting evidence on how IAA impacts the early regeneration process from germination to seedling emergence is sparse at best and focuses on the model plant *A. thaliana*. At high temperatures (29°C) these seedlings exhibit dramatic hypocotyl elongation compared with seedlings grown at 20°C (Gray et al., 1998). We also detected a corresponding increase in the level of IAA in *Ficus* seedlings grown at high temperatures, suggesting that temperature regulates auxin synthesis or catabolism to mediate this growth response.

Some of our results supported our fourth hypothesis but the expression of this phytohormone between treatments and growth forms was complex. The SA response of the non-hemiepiphytic *Ficus* species to temperature stress showed that they were more sensitive to temperature stress resulting in significantly lower SA values during seed imbibition but no significant effect was seen when stressed during germination. This pattern was reversed with increased SA concentrations when heat stress was received during emergence. This contrasts with the results observed in *A. thaliana* where seed germination and the seedling establishment was accompanied by an increase in SA (Alonso-Ramírez et al., 2009) but it is consistent with the inhibitory effect of SA observed in the *A. thaliana* and barley and maze germination (Guan and Scandalios, 1995; Rajjou et al., 2006; Xie et al., 2007). In plants, many hormones may interact to provide a complex response. For example, cross-talk between ABA and SA signaling can result in increased synthesis of ABA-regulated proteins, such as late embryogenesis abundant proteins, dehydrins, and heat shock proteins, which provide resilience to HTS (Rajjou et al., 2006). Our results where the regeneration stage had specific phytohormonal responses are consistent with the idea that the phytohormone effect on growth depends on the plant species and developmental stage (Rivas-San Vicente and Plasencia, 2011).

Other studies have shown that phytohormone can influence the temperature stress response in *Ficus* plants as a regulator of plant growth and development, and mediate the damage caused by stress. For example, brassinosteroids can alleviate the injury in *Ficus concinna* seedlings caused by high temperature by increasing antioxidant defense and sustaining glyoxalase systems (Jin et al., 2015). Our results are consistent with previous studies on the different bio-physiological mechanisms in the two growth forms (Hao et al., 2010, 2013; Chen et al., 2021). In *Ficus* species, the optimal maximum temperature for germination rises gradually during the summer in subtropical environments and optimum temperatures are present year-round in the tropics, which is consistent with the flowering and fruit setting observed in subtropical to tropical environments where the majority of the *Ficus* species are found. While germination is enhanced as temperature increases, seedling emergence and survival is repressed by temperatures higher than the optimal conditions (Castillo-Díaz et al., 2021; Chen et al., 2021). The distribution of these species within the tropical to the subtropical range is limited in higher elevations where autumn and nighttime temperatures fall below the upper limit for germination conditions (Castillo-Díaz et al., 2021; Chen et al., 2021). Therefore, the seed sensitivity to temperature is an ecologically important role in the detection of and responding to the appropriate timing for germination under natural conditions (Yoshioka et al., 1998; Baskin and Baskin, 2014b). Our study shows that the temperature stress experienced as transient extreme events may be modulated by phytohormone expression providing greater resilience to temperature stress in hemiepiphytic species, which had greater survival under all HTS treatments.

Plant regeneration is more sensitive than vegetative growth to many environmental stresses. Although HTS is becoming an increasingly more prevalent and common stressor due to recent global warming much of its effects are studied in crop species. Our study highlights the importance of understanding how HTS affects the early regeneration of ecologically important flora such as the keystone species in the *Ficus* genus. In our study both hemiepiphytic and non-hemiepiphytic the seeds of *Ficus* species were more sensitive to temperature stress when stress is experienced early in the regeneration process but with no effect when received after emergence. Further, seedling survival favored hemiepiphytes regardless under HTS received during imbibition, germination as well as seedling emergence which was modulated by phytohormones in a species- and regeneration stage-specific manner. The different eco-physiological mechanisms between the hemiepiphytic and non-hemiepiphytic species in *Ficus* make this group a good comparative model for understanding the patterns of subtropical and tropical tree species response to climate change. We conclude that the sensitivity to HTS is an ecologically important functional trait for detecting and responding to the appropriate timing for seed germination and seedling development. In the future, the *Ficus* study still needs to focus more on their bio-physiological mechanism, plant and seed anatomy, and molecular research to elucidate the relationships among plant and seed structure, physiological mechanism, genes expression, and abiotic stress.

## Data Availability Statement

The dataset presented in this study is available in the online Figshare Digital Repository, which can be accessed in the following link: https://doi.org/10.6084/m9.figshare.16851007.v1.

## Author Contributions

CF: conceptualization, methodology, investigation, formal analysis, and writing of the original draft. HC: conceptualization, methodology, formal analysis, and writing with review and editing. DC-D, BW, and K-FC: methodology and writing with review and editing. UG: conceptualization, methodology, formal analysis, supervision, funding acquisition, and writing with review and editing. All authors contributed to the article and approved the submitted version.

## Conflict of Interest

The authors declare that the research was conducted in the absence of any commercial or financial relationships that could be construed as a potential conflict of interest.

## Publisher’s Note

All claims expressed in this article are solely those of the authors and do not necessarily represent those of their affiliated organizations, or those of the publisher, the editors and the reviewers. Any product that may be evaluated in this article, or claim that may be made by its manufacturer, is not guaranteed or endorsed by the publisher.
