# Supplementary material for: Regeneration and Endogenous Phytohormone Responses to High-Temperature Stress Drive Recruitment Success in Hemiepiphytic Fig Species
Source: Front Plant Sci. 2021 Nov 29;12:754207. doi: 10.3389/fpls.2021.754207 (PMC8666629; doi:10.3389/fpls.2021.754207)
Supplement: Supplementary file 1 [file Data_Sheet_1.docx]

**Regeneration and Endogenous Phytohormone Responses to High-Temperature Stress Drive Recruitment Success in Hemiepiphytic Fig Species**

Chuangwei Fang^1^, Huayang Chen^1,2,3,4^, Diana Castillo-Díaz^1,2,4^, Bin Wen^5^, Kun-Fang Cao^1,2^, Uromi Manage Goodale^1,2,4*^

^1^Guangxi Key Laboratory of Forestry Ecology and Conservation, College of Forestry, Guangxi University, Daxuedonglu 100, Nanning, Guangxi 530004, China

^2^State Key Laboratory of Conservation and Utilization of Subtropical Agro-bioresources, College of Forestry, Guangxi University, Daxuedonglu 100, Nanning, Guangxi 530004, China

^3^State Key Laboratory of Vegetation and Environmental Change, Institute of Botany, The Chinese Academy of Sciences, Beijing 100093, P.R. China

^4^Seed Conservation Specialist Group, Species Survival Commission, International Union for Conservation of Nature, Gland 281196, Switzerland

^5^Center for Integrative Conservation, Xishuangbanna Tropical Botanical Garden, Chinese Academy of Sciences, Menglun, Mengla 666303, Yunnan, China

Running head: Ecophysiology of hemiepiphytism in *Ficus*

* Author for correspondence: UMG: uromi.manage.goodale@outlook.com; uromi.goodale@aya.yale.edu

**Keywords**: heat stress, *Ficus*, plant hormones, seed and seedling, climate change.


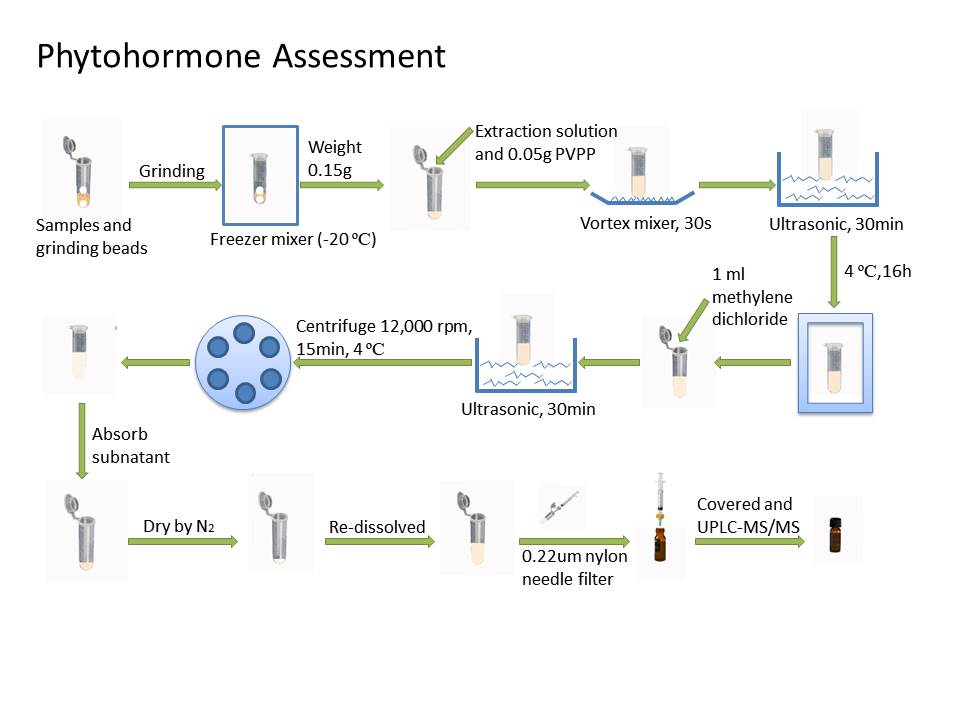


Supplementary Figure 1. Extraction and quantification procedures of phytohormones. N_2_, nitrogen; PVPP, polyvinylpolypyrrolidone.


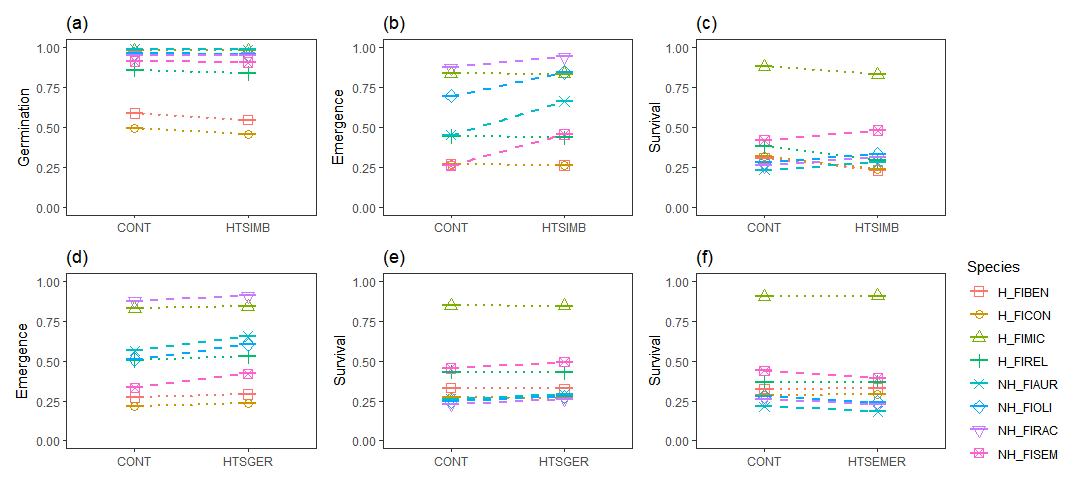


Supplementary Figure 2. Early regeneration responses in two growth forms (H = hemiepiphytic and NH = non-hemiepiphytic) of eight *Ficus* species (Table 1) high temperature stress (HTS) applied at different regeneration stages: seed imbibition (HTSIMB), seed germination (HTSGER), and seedling emergence (HTSEMER). The HTS was applied as 40ºC for 8 h from 10:00 am to 6:00 pm and the respective control treatment (CONT) was not given a HTS treatment. The responses to HTSIMB were measured as the probability of seed germination (a), probability of seedling emergence (b) and probability of seedling survival (c). The responses to HTSGER were measured as the probability of seedling emergence (d) and the probability of seedling survival (e). The response to HTSEMER was measured as the probability of seedling survival (f).


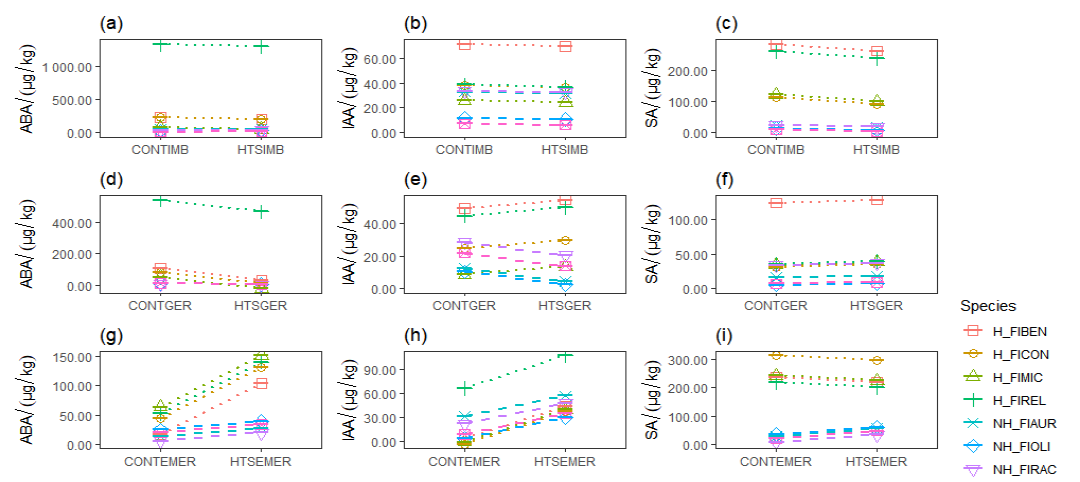


Supplementary Figure 3. The concentrations of three endogenous phytohormones, abscisic acid (ABA), indole-3-acetic acid (IAA), and salicylic acid (SA), in two growth forms (H = hemiepiphytic and NH = non-hemiepiphytic) of eight *Ficus* species (Table 1) when high temperature stress (HTS) was applied at different regeneration stages: seed imbibition (HTSIMB), seed germination (HTSGER), and seedling emergence (HTSEMER). The HTS was applied as 40ºC for 8 h from 10:00 am to 6:00 pm and the respective control treatment (CONT) was not given a HTS treatment. The concentration of ABA, IAA, and SA after HTSIMB and for their control treatments are given in graphs a-c, respectively. The concentration of ABA, IAA, and SA after HTSGER and for their control treatments are given in graphs d-f, respectively. The concentration of ABA, IAA, and SA after HTSGER and for their control treatments are given in graphs g-i, respectively.

Supplementary Table 1. Settings of the ultra-performance liquid chromatography. All chemicals are HPLC grade.

| Chromatographic Conditions | | | | | | |
| --- | --- | --- | --- | --- | --- | --- |
| Chromatographic Column | Column Temperature | Flow Rate | Injection Volume | Mobile Phase | | |
| Waters ACQUITY UPLC BEH C18 | 40 ºC | 0.3 mL/min | 2 μL | A | 0.1% formic acid | |
|  |  |  |  | B | | methanol |

Supplementary Table 2. Information of the chromatography gradient elution procedure. All chemicals are HPLC grade.

| Chromatography Gradient Elution Procedure | | | |
| --- | --- | --- | --- |
| Time  (min) | Flow Rate (mL/min) | Mobile phase A (%) | Mobile phase B  (%) |
| 0 | 0.3 | 80 | 20 |
| 6 | 0.3 | 50 | 50 |
| 8 | 0.3 | 0 | 100 |
| 10 | 0.3 | 0 | 100 |
| 10.1 | 0.3 | 80 | 20 |
| 13.0 | 0.3 | 80 | 20 |

Supplementary Table 3. Ion pair of abscisic acid (ABA), indole-3-acetic acid (IAA), salicylic acid (SA) and parameters settings of the tandem mass spectrometry. All chemicals are HPLC grade.

| MS/MS Parameters | | | | | |
| --- | --- | --- | --- | --- | --- |
| Phytohormone | Precursor ion (m/z) | Product ion (m/z) | Dell time (s) | Cone  Voltage (V) | Collision energy (eV) |
| ABA | 263.10 | 219.07 | 0.2 | 40 | 10 |
| ABA | 263.10 | 153.09 | 0.2 | 40 | 10 |
| IAA | 176.10 | 130.10 | 0.2 | 40 | 15 |
| IAA | 176.10 | 103.00 | 0.2 | 40 | 20 |
| SA | 137.11 | 92.80 | 0.2 | 40 | 24 |
| SA | 137.11 | 64.80 | 0.2 | 40 | 26 |

Supplementary Table 4. Linear equation and correlation coefficients of standard phytohormones; abscisic acid (ABA), indole-3-acetic acid (IAA), salicylic acid (SA). All chemicals are HPLC grade.

| Linear Equation and Correlation Coefficients of Three Phytohormones | | |
| --- | --- | --- |
| Phytohormone | Linear Equation | R^2 |
| ABA | y = 18068.2558x - 2780.2930 | 0.9991 |
| IAA | y = 37235.5040x + 18620.5768 | 0.9997 |
| SA | y = 11948.7300x + 1253.6529 | 0.999 |
